# Supplementary material for: Analysis of PPARγ Signaling Activity in Psoriasis
Source: Int J Mol Sci. 2021 Aug 10;22(16):8603. doi: 10.3390/ijms22168603 (PMC8395241; doi:10.3390/ijms22168603)
Supplement: Supplementary file 1 [file ijms-22-08603-s001.zip › Supplemental materials_Analysis of PPARg signaling activity in psoriasis/Pathway models/Models images and html files/Differentiation of psoriatic T cells/86935.html]

IL17F


# Protein IL17F

|  |  |
| --- | --- |
| URN | urn:agi-llid:112744 |
| Total Entities | 0 |
| Connectivity | 1228 |
| Name | IL17F |
| Description | interleukin 17F |
| Notes | The protein encoded by this gene is a cytokine that shares sequence similarity with IL17. This cytokine is expressed by activated T cells, and has been shown to stimulate the production of several other cytokines, including IL6, IL8, and CSF2/GM\_CSF. This cytokine is also found to inhibit the angiogenesis of endothelial cells and induce endothelial cells to produce IL2, TGFB1/TGFB, and monocyte chemoattractant protein-1. [provided by RefSeq, Jul 2008] |
| Primary Cell Localization | Extracellular |
| Class | Ligand |

---

|  |  |
| --- | --- |
| Pathway | AHR Signaling in Th17 Cells Function |
|  | Th17-Cell Differentiation |
|  | Lymphocyte Mediated Myocardial Injury in Myocarditis |
|  | Proteins Involved in Myocarditis |
|  | IL17 Signaling in Psoriasis |
|  | Proteins Involved in Psoriasis |
|  | T-Cells Differentiation Block in Psoriasis |
|  | Th17-Cell Function in Systemic Lupus Erythematosus |
|  | Th17-Cell Activation in Crohn's Disease |
|  | Il17 Signaling Related Neutrophilia in Asthma |
|  | IL17F Signaling in Bronchial Epithelial Cell in Asthma |
|  | Th17-Cell Differentiation in Asthma |
|  | Th17-Cell and Th1 Immune Responsein Psoriatic Arthritis |
|  | Synovial Fibroblast Activation in Psoriatic Arthritis |
|  | Keratinocyte Activation in Psoriatic Arthritis |
|  | Th17-Cell Numbers Reduction in HIV |
|  | Proteins Involved in Endometriosis |
|  | Cytokines Trigger Otitis Media |
|  | Proteins with Altered Expression in Asthma |
|  | IL21 Expression Targets |
|  | PPAR Psoriasis |
|  | pprarg neg, uknown targets, ps-positive |
|  | prarg neg,ukn expres targets, ps-positive |
|  | PPARG negative targets |
|  | pparg expr target\_regulators |
|  | Neighbors of disease exacerbation |
|  | Neighbors of tumor progression |
|  | Neighbors of cancer progression |
|  | Neighbors of inflammatory status |
|  | Neighbors of overall survival |
|  | Neighbors of disease severity |
|  | Neighbors of tumor size |
|  | Neighbors of bacterial load |
|  | Neighbors of lethality |
|  | Neighbors of disease-free survival |
|  | Neighbors of macrophage count |
|  | Neighbors of microvessel density |
|  | Neighbors of neutrophil count |
|  | Neighbors of bone mineral density |
|  | Neighbors of cancer risk |
|  | Neighbors of gastric cancer risk |
|  | Neighbors of insulin level |
|  | Neighbors of disease exacerbation |
|  | Neighbors of tumor progression |
|  | Neighbors of cancer progression |
|  | Neighbors of inflammatory status |
|  | Neighbors of overall survival |
|  | Neighbors of disease severity |
|  | Neighbors of tumor size |
|  | Neighbors of bacterial load |
|  | Neighbors of lethality |
|  | Neighbors of disease-free survival |
|  | Neighbors of macrophage count |
|  | Neighbors of microvessel density |
|  | Neighbors of neutrophil count |
|  | Neighbors of bone mineral density |
|  | Neighbors of cancer risk |
|  | Neighbors of gastric cancer risk |
|  | Neighbors of insulin level |
|  | Neighbors of cell development |
|  | Neighbors of immune response |
|  | Neighbors of inflammatory response |
|  | Neighbors of wound healing |
|  | Neighbors of angiogenesis |
|  | Neighbors of innate immune response |
|  | Neighbors of cell function |
|  | Neighbors of macrophage activation |
|  | Neighbors of cell formation |
|  | Neighbors of adaptive immune response |
|  | Neighbors of osteoclast development |
|  | Neighbors of epithelial to mesenchymal transition |
|  | Neighbors of immunity |
|  | Neighbors of chemotaxis |
|  | Neighbors of osteoclast differentiation |
|  | Neighbors of tissue remodeling |
|  | Neighbors of T-cell activation |
|  | Neighbors of tumor growth |
|  | Neighbors of tissue repair |
|  | Neighbors of cell infiltration |
|  | Neighbors of SMC proliferation |
|  | Neighbors of lumen formation |
|  | Neighbors of adipogenesis |
|  | Neighbors of pregnancy |
|  | Neighbors of cell motility |
|  | Neighbors of bone resorption |
|  | Neighbors of fibroblast proliferation |
|  | Neighbors of adipocyte differentiation |
|  | Neighbors of neutrophil recruitment |
|  | Neighbors of cell migration |
|  | Neighbors of cancer growth |
|  | Neighbors of stem cell proliferation |
|  | Neighbors of smooth muscle cell migration |
|  | Neighbors of monocyte recruitment |
|  | Neighbors of cell invasion |
|  | Neighbors of immune system function |
|  | Neighbors of autophagy |
|  | Neighbors of cell transdifferentiation |
|  | Neighbors of cell growth |
|  | Neighbors of sensitization |
|  | Neighbors of cellular immune response |
|  | Neighbors of cell differentiation |
|  | Neighbors of keratinocyte proliferation |
|  | Neighbors of psoriasis |
|  | Neighbors of inflammatory disease |
|  | Neighbors of rheumatoid arthritis |
|  | Neighbors of fibrosis |
|  | Neighbors of colitis |
|  | Neighbors of arthritis |
|  | Neighbors of inflammatory bowel disease |
|  | Neighbors of injury |
|  | Neighbors of metastasis |
|  | Neighbors of carcinogenesis |
|  | Neighbors of multiple sclerosis |
|  | Neighbors of chronic inflammation |
|  | Neighbors of inflammation |
|  | Neighbors of pathological angiogenesis |
|  | Neighbors of obesity |
|  | Neighbors of diabetes mellitus |
|  | Neighbors of asthma |
|  | Neighbors of pneumonia |
|  | Neighbors of hyperplasia |
|  | Neighbors of liver fibrosis |
|  | Neighbors of chronic obstructive pulmonary disease |
|  | Neighbors of myocardial infarction |
|  | Neighbors of leukocyte infiltration |
|  | Neighbors of airway inflammation |
|  | Neighbors of infection |
|  | Neighbors of renal injury |
|  | Neighbors of macrophage infiltration |
|  | Neighbors of sepsis |
|  | Neighbors of encephalomyelitis |
|  | Neighbors of autoimmune disease |
|  | Neighbors of dermatitis |
|  | Neighbors of neutrophil infiltration |
|  | Neighbors of diabetic nephropathy |
|  | Neighbors of immunopathology |
|  | Neighbors of systemic sclerosis |
|  | Neighbors of hepatocellular carcinoma |
|  | Neighbors of autoimmunity |
|  | Neighbors of gastric cancer |
|  | Neighbors of experimental autoimmune encephalomyelitis |
|  | Neighbors of colorectal cancer |
|  | Neighbors of breast cancer |
|  | Neighbors of collagen-induced arthritis |
|  | Neighbors of cartilage degeneration |
|  | Neighbors of kidney disease |
|  | Neighbors of lung disease |
|  | Neighbors of cancer |
|  | Neighbors of neoplasm |
|  | Neighbors of endometriosis |
|  | Neighbors of respiratory hypersensitivity |
|  | Neighbors of synovitis |
|  | Neighbors of hypertrophy |
|  | Neighbors of proteinuria |
|  | Neighbors of epithelial cell |
|  | Neighbors of monocyte |
|  | Neighbors of macrophage |
|  | Neighbors of PBMC |
|  | Neighbors of pneumocyte |
|  | Neighbors of mononuclear cell |
|  | Neighbors of inflammatory cell |
|  | Neighbors of neutrophil |
|  | Neighbors of activated T-cell |
|  | Neighbors of immunocompetent cell |
|  | Neighbors of granulocyte |
|  | Neighbors of B-cell |
|  | Neighbors of leukocyte |
|  | Neighbors of T-cell |
|  | Neighbors of mast cell |
|  | Neighbors of spleen cell |
|  | Neighbors of hematopoietic cell |
|  | Neighbors of lymphocyte |
|  | Neighbors of thymic epithelial cell |
|  | Neighbors of lymphoid cell |
|  | Neighbors of blood cell |
|  | Neighbors of lymph node cell |
|  | Neighbors of kidney |
|  | Neighbors of colon |
|  | Neighbors of synovial membrane |
|  | Neighbors of stomach |
|  | Neighbors of islets of Langerhans |
|  | Neighbors of peritoneum |
|  | Neighbors of NF-kB family |
|  | Neighbors of IL1 family |
|  | Neighbors of PI3K |
|  | Neighbors of STAT family |
|  | Neighbors of IFNAR ligand |
|  | Neighbors of interleukin |
|  | Neighbors of JAK |
|  | Neighbors of IL23 |
|  | Neighbors of IL1R |
|  | Neighbors of IL12 |
|  | Neighbors of STAT5 |
|  | Neighbors of retinoid-X receptor subfamily |
|  | Neighbors of RA receptor |
|  | Neighbors of TLR4/Myd88 |
|  | comon ps\_pos, pprarg\_neg targets |
|  | Model of PPARG signaling in psoriasis |
|  | PPARG negative regulators and targets |
|  | Model of PPARG related pathways in psoriasis (short version) |
|  | New Pathway (3) |
|  | 1\_Differentiation of psoriatic T cells |
|  | 2\_Interleukin-17 and interleukin-22 signaling in psoriasis |
|  | 3\_2\_Regulation of airway surface liquid (ASL) thickness |
|  | 2\_3\_Eosinophilia and neutrophilia in asthma: Neutrophil chemotaxis and activation |
|  | 4\_2\_Airway smooth muscle cells proliferation and airway tissue remodeling |
|  | 1\_Defects in response to pathogens in the gut promotes inflammation in Crohn's disease |
|  | 4\_1\_Polymorphisms associated with inflammatory bowel diseases |
|  | Model of PPARG signaling in psoriais (tested) |
|  | Differentiation of psoriatic T cells |
|  | Interleukin-17 and interleukin-22 signaling in psoriasis |

---

|  |  |
| --- | --- |
| MedScan ID | 112744 |

---

|  |  |
| --- | --- |
| LocusLink ID | 112744 |
|  | 257630 |
|  | 301291 |

---

|  |  |
| --- | --- |
| Alias | cytokine ML I |
|  | interleukin IL17F |
|  | interleukin 17F |
|  | MGC114402 |
|  | cytokine ML-1 |
|  | IL17F |
|  | CANDF6 |
|  | ML-1 |
|  | C87042 |
|  | ML1 |
|  | IL-17F |
|  | interleukin-17F |

---

|  |  |
| --- | --- |
| GO ID | 0005125 |
|  | 0019955 |
|  | 0005126 |
|  | 0042803 |
|  | 0051216 |
|  | 0042089 |
|  | 0019221 |
|  | 0006954 |
|  | 0097400 |
|  | 0042109 |
|  | 0016525 |
|  | 1900017 |
|  | 2000778 |
|  | 0045944 |
|  | 0045423 |
|  | 0045076 |
|  | 0045408 |
|  | 0045414 |
|  | 0017015 |
|  | 0005576 |
|  | 0005615 |
|  | 0010469 |
|  | 0007166 |
|  | 0006029 |

---

|  |  |
| --- | --- |
| KEGG ID | hsa:112744 |
|  | mmu:257630 |
|  | rno:301291 |

---

|  |  |
| --- | --- |
| Organism | Homo sapiens {Organism urn:agi-taxid:9606} |
|  | Mus musculus {Organism urn:agi-taxid:10090} |
|  | Rattus norvegicus {Organism urn:agi-taxid:10116} |
|  | Mus musculus |
|  | Homo sapiens |
|  | Rattus norvegicus |

---

|  |  |
| --- | --- |
| Mouse chromosome position | 1 |

---

|  |  |
| --- | --- |
| OMIM ID | 606496 |
|  | 613956 |

---

|  |  |
| --- | --- |
| Rat chromosome position | 9q13 |

---

|  |  |
| --- | --- |
| Hugo ID | 16404 |
|  | HGNC:16404 |

---

|  |  |
| --- | --- |
| Human chromosome position | 6p12.2 |
|  | 6p12 |

---

|  |  |
| --- | --- |
| Swiss-Prot Accession | Q96PD4 |
|  | Q96PD4.3 |
|  | Q7TNI7 |
|  | Q7TNI7.1 |
|  | Q5BJ95 |
|  | Q5BJ95.2 |
|  | Q6NSI0 |
|  | Q7Z6P4 |
|  | Q96PI8 |
|  | Q9NUE6 |
|  | Q8K4C3 |

---

|  |  |
| --- | --- |
| GenBank ID | NC\_000006 |
|  | XM\_011514276 |
|  | XP\_011512578 |
|  | NM\_052872 |
|  | NP\_443104 |
|  | NG\_031869 |
|  | AL034343 |
|  | CH471081 |
|  | EAX04363 |
|  | EAX04364 |
|  | CS399130 |
|  | CAL44888 |
|  | GM832011 |
|  | CAV28581 |
|  | JA104581 |
|  | CCA63960 |
|  | JA381711 |
|  | CCB07362 |
|  | AF332389 |
|  | AAL14427 |
|  | AF384857 |
|  | AAK83350 |
|  | BC070124 |
|  | AAH70124 |
|  | JF305974 |
|  | ADY18335 |
|  | Q96PD4 |
|  | NC\_000067 |
|  | NM\_145856 |
|  | NP\_665855 |
|  | XM\_006495523 |
|  | XP\_006495586 |
|  | AC159614 |
|  | CH466536 |
|  | EDL14379 |
|  | GM832044 |
|  | CAV28592 |
|  | AB116259 |
|  | BAC81535 |
|  | AF458064 |
|  | AAM77568 |
|  | AK157101 |
|  | BAE33962 |
|  | AY380822 |
|  | AAQ88439 |
|  | BC145977 |
|  | Q7TNI7 |
|  | NC\_005108 |
|  | NM\_001015011 |
|  | NP\_001015011 |
|  | AC\_000077 |
|  | AABR07067030 |
|  | AAHX01058668 |
|  | CH473987 |
|  | EDM18651 |
|  | BC091568 |
|  | AAH91568 |
|  | Q5BJ95 |
|  | NC\_018917 |
|  | AMYH02013730 |
|  | XM\_006495522 |
|  | XP\_006495585 |
|  | AC\_000023 |
|  | AAHY01004447 |
|  | BC160259 |
|  | AAI60259 |
|  | AC\_000138 |
|  | ABBA01050558 |

---

|  |  |
| --- | --- |
| Swiss-Prot ID | IL17F\_HUMAN |
|  | IL17F\_MOUSE |
|  | IL17F\_RAT |

---

|  |  |
| --- | --- |
| Cell Localization | Secreted |

---

|  |  |
| --- | --- |
| Ensembl ID | ENSG00000112116 |
|  | ENSP00000337432.4 |
|  | ENST00000336123.4 |
|  | ENSMUSG00000041872 |
|  | ENSMUSP00000046960.3 |
|  | ENSMUST00000039046.9 |
|  | ENSMUSP00000140122.1 |
|  | ENSMUST00000189301.1 |
|  | ENSRNOG00000012509 |
|  | ENSRNOP00000016717.6 |
|  | ENSRNOT00000016717.5 |
|  | ENSP00000337432 |
|  | ENST00000336123 |
|  | ENSMUSP00000046960 |
|  | ENSMUST00000039046 |
|  | ENSMUSP00000140122 |
|  | ENSMUST00000189301 |
|  | ENSRNOP00000016717 |
|  | ENSRNOT00000016717 |

---

|  |  |
| --- | --- |
| MGI ID | MGI:2676631 |

---

|  |  |
| --- | --- |
| RGD ID | 1311398 |

---

|  |  |
| --- | --- |
| Unigene ID | Hs.272295 |
|  | Mm.222807 |
|  | Rn.34022 |

---

|  |  |
| --- | --- |
| Homologene ID | 17115 |

---

|  |  |
| --- | --- |
| Shape | Rhomb |

---
